# A Case of Conjunctival Amyloidosis with Repeated Subconjunctival Hemorrhage

**DOI:** 10.1155/2017/5423027

**Published:** 2017-02-23

**Authors:** Takaaki Ando, Toshiyuki Oshitari, Mamiko Saito, Ayako Tawada, Takayuki Baba, Jiro Yotsukura, Shuichi Yamamoto

**Affiliations:** Department of Ophthalmology and Visual Science, Chiba University Graduate School of Medicine, Chiba, Japan

## Abstract

Conjunctival amyloidosis is a very rare disease, and its presence may be a sign of systemic amyloidosis. We present our ocular and systemic findings in a patient with conjunctival amyloidosis. A 43-year-old man had repeated subconjunctival hemorrhages (SCHs) for two years and was referred to the Chiba University Hospital. He had comprehensive ophthalmological and systemic examinations to determine the cause of the SCHs. His visual acuities were 1.2 OU, and the intraocular pressures were 13-14 mmHg OU. Magnetic resonance imaging was normal. Initially, the SCH was the only abnormality. After 3 months, the SCH had partially cleared, and a pink mass was detected in the superior area of the subconjunctiva. Partial biopsy and histopathological examinations showed a greenish birefringence and dichroism under polarized light illumination. The birefringence was located in amyloid fibers. Immunofixation electrophoresis detected *λ*-light chain abnormality in the ocular biopsy specimen but systemic examinations did not find any lesions. Multiple myeloma was ruled out, and the patient is being followed closely to detect any early signs of systemic amyloidosis. Because repeated SCHs might be initial signs of systemic amyloidosis, patients with conjunctival amyloidosis should be comprehensively examined for systemic amyloidosis because of its poor life prognosis.

## 1. Introduction

Amyloidosis disorders are characterized by accumulation of insoluble fibrillar proteins known as amyloids in many organs and tissues throughout the body [1]. Amyloidosis is classified into local and systemic disorders depending on the location and extent of the disease, or secondary for the acquired forms [1]. Because systemic amyloidosis is a life-threatening disease, systemic involvement must be ruled out in cases of localized amyloidosis as in conjunctival amyloidosis.

Conjunctival amyloidosis is very rare. An earlier pathological study of 2,455 cases of conjunctival lesions showed that conjunctival amyloidosis was diagnosed in only 5 patients (0.002%) [2]. In addition, most of the conjunctival amyloidosis was found to be localized amyloidosis. A PubMed search extracted only six cases of systemic amyloidosis accompanied by conjunctival amyloidosis [3–8]. Over 50 cases of conjunctival amyloidosis have been reported in PubMed, but most of these were localized amyloidosis [9, 10]. Thus, conjunctival amyloidosis accompanied by systemic amyloidosis is extremely rare.

The previous review indicates that, of the 50 patients with conjunctival amyloidosis, 84% of patients have conjunctival mass and 33% of patients have subconjunctival hemorrhage [9]. Thus, in many cases, the initial diagnoses were lacrimal gland tumor, allergic conjunctivitis, lymphoma, or subconjunctival hemorrhage. In some cases, recurrence of subconjunctival hemorrhage was the initial signs of the conjunctival amyloidosis [11].

We report a case of conjunctival amyloidosis with repeated subconjunctival hemorrhages.

## 2. Case Report

A 43-year-old man had repeated subconjunctival hemorrhages for two years and was referred to the Chiba University Hospital for further examinations in May 2015. He had a history of IgA nephropathy but had no family history of amyloidosis. At the initial ophthalmological examinations, his visual acuities were 1.2 OU, and the intraocular pressures were 13-14 mmHg OU. Slit-lamp examinations showed that the anterior chamber and the lens were clear in both eyes. Fundus examinations showed central serous chorioretinopathy in the right eye. Magnetic resonance imaging indicated that the eye, orbit, and the brain were normal.

**Figure 1:**
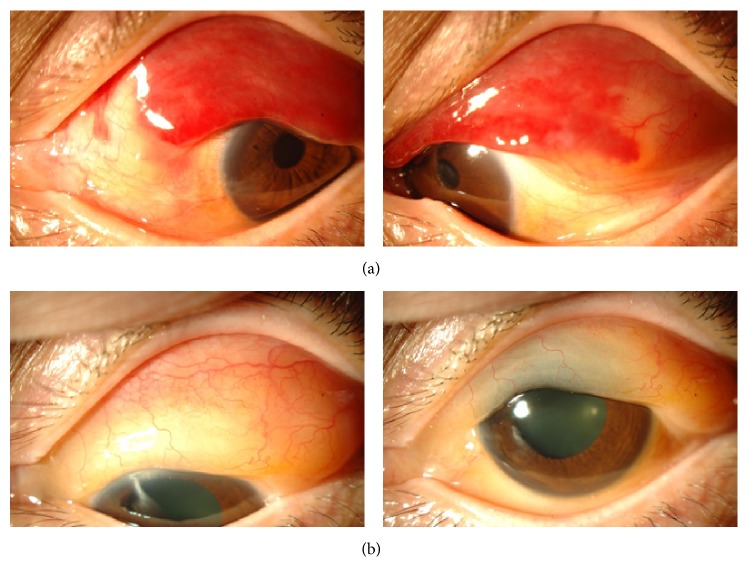
Slit-lamp photographs of anterior segment at the initial visit (a) and three months after the first visit (b). At the initial visit, subconjunctival hemorrhages masked a mass. Three months later, a salmon-pink mass was detected in the superior area of the subconjunctiva.

**Figure 2:**
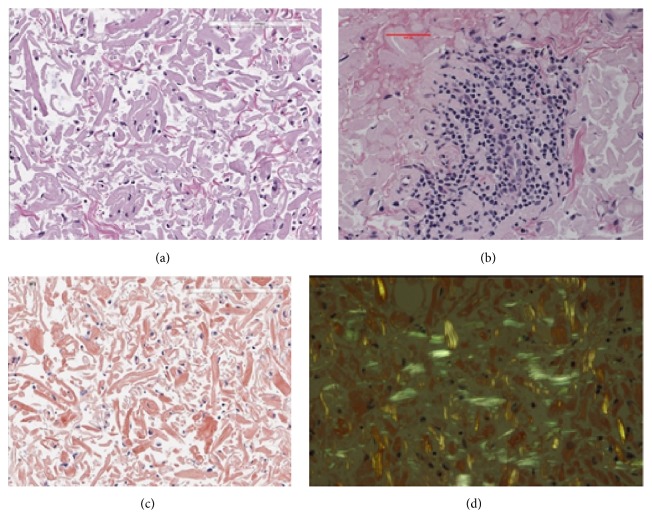
Representative histopathologic findings in the biopsy specimen of the mass in the subconjunctiva. (b) High magnification of H-E stained section showing an infiltration of inflammatory cells. (a) Lower magnification of H&E stained section showing an accumulation of amorphous, acellular eosinophilic material in the substantia propria of the conjunctiva. (c) Accumulated materials are direct fast scarlet (DFS) positive. (d) Polarized microscopy shows green birefringence and dichroism of amyloid fibers.

**Figure 3:**
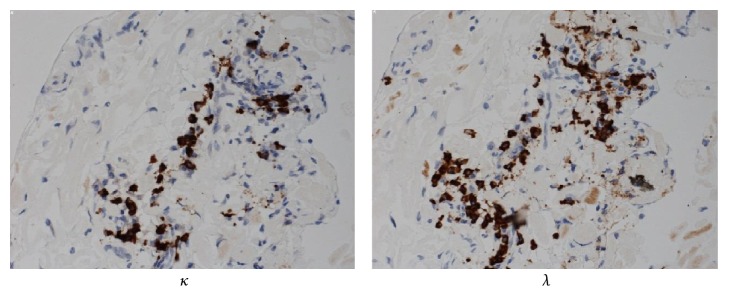
In situ hybridization of immunoglobulin light chains of the specimens. Both *λ* and *κ* chains subunits were strongly detected in the infiltrated inflammatory cells.

**Figure 4:**
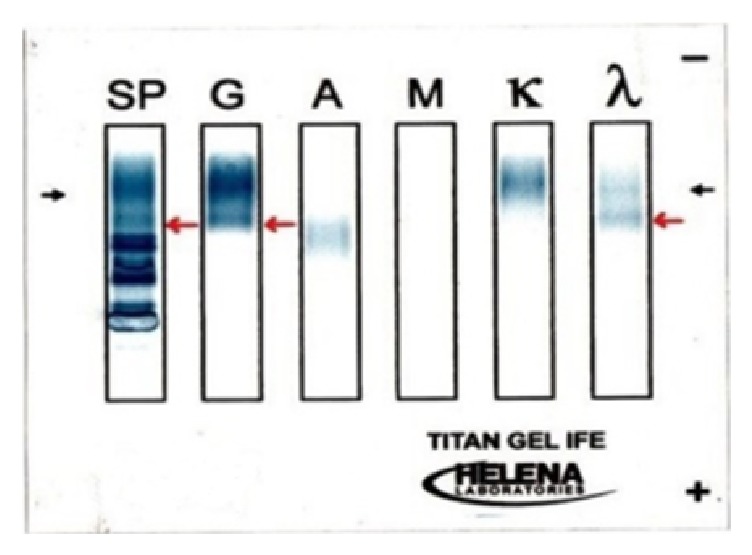
Results of immunofixation electrophoresis of the serum. Abnormalities of the *λ*-light chain are present (red arrows). SP is the total proteins. G is the IgG, A is the IgA, and M is the IgM.

**Figure 5:**
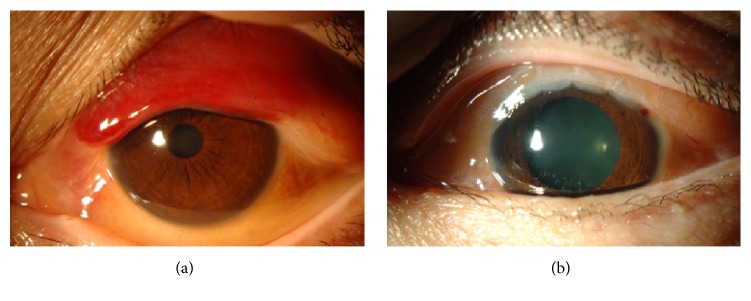
Slit-lamp photographs before and three months after surgery. (b) After surgery, the subconjunctival hemorrhage was not observed. (a) The preoperative subconjunctival hemorrhage can be seen.

Initially, the subconjunctival hemorrhages were the only abnormality (see Supplemental Figure in Supplementary Material available online at https://doi.org/10.1155/2017/5423027). Three months later, the subconjunctival hemorrhage had partially cleared and a pink mass was detected in the superior area of the subconjunctiva (Figure 1). The subconjunctival hemorrhage was accompanied with chemosis and overlapping the superior cornea. These findings are enough to suspect any abnormalities like existence of mass or inflammation. Thus, we initially suspected a malignant lymphoma and performed partial biopsy of the mass. During the surgery, we noted that Tenon's capsule was rich in blood vessels and hemorrhages. The areas with hemorrhages were completely cauterized for hemostasis. Histopathological examinations of the biopsy specimen showed amyloid fibers that appeared greenish under polarized light illumination (Figure 2). This appearance was due to birefringence and dichroism. In situ hybridization detected both *λ* and *κ* chains in the infiltrated inflammatory cells (Figure 3). A diagnosis of conjunctival amyloidosis was made. Blood tests found increases of both free *λ* and *κ* chains but the immunoglobulin *λ*/*κ* free light chains ratio was normal. Immunofixation electrophoresis detected *λ*-light chain abnormality (Figure 4) but systemic examinations did not find any in the heart and the gastrointestine. Renal biopsy could not be performed because of renal atrophy. Although multiple myeloma was ruled out, the patient is being followed carefully to detect any signs of systemic amyloidosis. The renal function is being followed in the Department of Nephrology. Although the subconjunctival hemorrhage did not recur after two months of biopsy, the temporal subconjunctival hemorrhage recurred a few times during a year after the biopsy (Figure 5).

## 3. Discussion

There is some evidence that repeated subconjunctival hemorrhages may be an early sign of conjunctival amyloidosis because amyloid infiltration into the walls of the conjunctival vessels can reduce their rigidity and lead to hemorrhages [3]. Thus, in cases of repeated subconjunctival hemorrhages, partial biopsy is recommended to make an early diagnosis of conjunctival amyloidosis.

Lee et al. previously presented the similar case of ours. They presented the case that repeated subconjunctival hemorrhage was an initial presentation of primary localized conjunctival amyloidosis [11]. They performed biopsy three times to reveal the presence of amyloid in the conjunctival tissue. Their patient had no systemic diseases but they discuss that amyloid in the walls of the blood vessels may be related to recurrence of subconjunctival hemorrhages [11].

Past studies have shown systemic amyloidosis rarely accompanied by conjunctival amyloidosis [3–8], and most cases of conjunctival amyloidosis are found to be localized amyloidosis. However, systemic amyloidosis is a life-threatening disease, and a complete systemic evaluation should be performed even in cases of conjunctival amyloidosis.

In our case, we did not make a final diagnosis of systemic amyloidosis but serum immunofixation electrophoresis detected *λ*-light chain abnormality. Thus, we could not rule out the possibility of multiple myeloma. In addition, his past history of IgA nephropathy was also suspicious. His renal function was not good; that is, serum creatinine was 3.3 mg/dL and e-GFR was 17.7 mL/min/1.73 m^2^. Therefore, careful follow-up examinations to detect an early depression of renal function and the development of multiple myeloma must be carried out because the previous report indicates that AL type of amyloidosis develops 15% of cases of multiple myeloma [12].

In conclusion, repeated SCH may be an initial sign of conjunctival amyloidosis. Patients with conjunctival amyloidosis should be examined comprehensively and regularly for systemic amyloidosis because of its poor life prognosis.

## Supplementary Material

Supplemental Figure. Slit-lamp photographs of both eyes at the primary eye clinic before the first visit of our hospital. The right eye showed no abnormalities. In the left eye, subconjunctival hemorrhage was observed with mild chemosis. Conjunctivochalasis causing repeated subconjunctival hemorrhages was not observed.

## References

[1] Merlini G., Bellotti V. (2003). Molecular mechanisms of amyloidosis. *New England Journal of Medicine*.

[2] Grossniklaus H. E., Green W. R., Luckenbach M., Chan C. C. (1987). Conjunctival lesions in adults. A clinical and histopathologic review. *Cornea*.

[3] Leibovitch I., Selva D., Goldberg R. A. (2006). Periocular and orbital amyloidosis. Clinical characteristics, management, and outcome. *Ophthalmology*.

[4] Purcell J. J., Birkenkamp R., Tsai C. C., Riner R. N. (1983). Conjunctival involvement in primary systemic nonfamilial amyloidosis. *American Journal of Ophthalmology*.

[5] Abdallah A.-O., Westfall C., Brown H., Muzaffar J., Atrash S., Nair B. (2012). Unilateral conjunctival AL kappa amyloidosis with trace evidence of systemic amyloidosis. *The American Journal of Case Reports*.

[6] Iijima S. (1992). Primary systemic amyloidosis: an unique case complaining of diffuse eyelid swelling and conjunctival involvement. *Journal of Dermatology*.

[7] Shields J. A., Eagle R. C., Shields C. L., Green M., Singh A. D. (2000). Systemic amyloidosis presenting as a mass of the conjunctival semilunar fold. *American Journal of Ophthalmology*.

[8] Correa L. J., Maccio J. P., Esposito E. (2015). Systemic amyloidosis with bilateral conjunctival involvement: a case report. *BMC Ophthalmology*.

[9] Demirci H., Shields C. L., Eagle R. C., Shields J. A. (2006). Conjunctival amyloidosis: report of six cases and review of the literature. *Survey of Ophthalmology*.

[10] Suesskind D., Ziemssen F., Rohrbach J. M. (2015). Conjunctival amyloidosis—clinical and histopathologic features. *Graefe's Archive for Clinical and Experimental Ophthalmology*.

[11] Lee H.-M., Naor J., Deangelis D., Rootman D. S. (2000). Primary localized conjunctival amyloidosis presenting with recurrence of subconjunctival hemorrhage. *American Journal of Ophthalmology*.

[12] Gertz M. A., Lacy M. Q., Dispenzieri A. (1999). Amyloidosis: recognition, confirmation, prognosis, and therapy. *Mayo Clinic Proceedings*.

