# Supplementary material for: A Case of Conjunctival Amyloidosis with Repeated Subconjunctival Hemorrhage
Source: Case Rep Ophthalmol Med. 2017 Feb 23;2017:5423027. doi: 10.1155/2017/5423027 (PMC5343237; doi:10.1155/2017/5423027)

Supplemental Figure. Slit-lamp photographs of both eyes at the primary eye clinic before the first visit of our hospital. The right eye showed no abnormalities. In the left eye, subconjunctival hemorrhage was observed with mild chemosis. Conjunctivochalasis causing repeated subconjunctival hemorrhages was not observed.

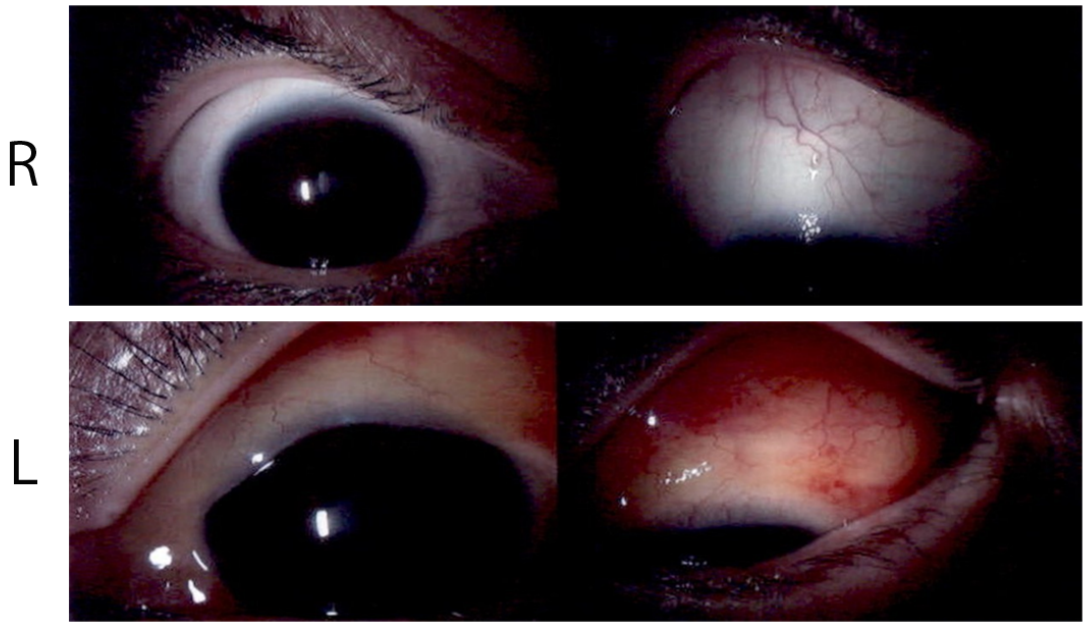

Supplement: Supplementary file 1 — Supplemental Figure. Slit-lamp photographs of both eyes at the primary eye clinic before the first visit of our hospital. The right eye showed no abnormalities. In the left eye, subconjunctival hemorrhage was observed with mild chemosis. Conjunctivochalasis causing repeated subconjunctival hemorrhages was not observed. [file 5423027.f1.pdf]
